# Psychometric evaluation of the Spanish version of the Pediatric Quality of Life Eosinophilic Esophagitis Questionnaire (Peds QL-EoE Module ™)

**DOI:** 10.1186/s12955-023-02211-0

**Published:** 2023-12-13

**Authors:** Ruth Garcia – Martinez de Bartolomé, Josefa Barrio-Torres, MLuz Cilleruelo- Pascual, Juan José Rodríguez-Soler, Ángel Gil-de Miguel, Tomás Sebastián-Viana, the Spanish Study Group of Children’s Eosinophilic Esophagitis Quality of Life, Víctor Vila-Miravet, Enrique La Orden-Izquierdo, Sonia Fernández-Fernández, Myriam Herrero-Álvarez, Marta Soria-López, Gonzalo Botija-Arcos, Alejandro Rodríguez-Martínez, Gonzalo Galicia-Poblet, Alejandro García-Díaz, Marta Herreros-Sáenz, Javier Blasco-Alonso, Gloria Rodrigo-García, Natalia Alonso-Pérez, Ana Fernández de Valderrama-Rodríguez, Noel Oppenau-López, Begoña Pérez-Moneo, Sara Feo-Ortega, Raquel Vecino-López, Paloma Donado-Palencia, José Ramón Alberto-Alonso, Margarita Revenga-Parra, Helena Lorenzo-Garrido, Miguel Ángel Carro-Rodríguez, Luis Grande-Herrero, Saioa Vicente-Santamaría, Elena Balmaseda-Serrano, María Carmen Miranda-Cid, Jessica Martín-González, Ruth García-Romero, Diana García-Tirado, Jana Rizo-Pascual, Pedro Alonso-López, Miriam Blanco-Rodríguez, Alicia Rendo-Vázquez, Antonio Millán-Jiménez, Ana Castro-Millán, Eduard Bastida-Ratera

**Affiliations:** 1 Department of Epidemiology and Public Health, Universidad Rey Juan Carlos, Madrid, Spain; 2 Department of Pediatrics, EAP Valle de La Oliva, Majadahonda, Madrid, Spain; 3 Department of Pediatrics, HU Fuenlabrada, Madrid, Spain; 4 Department of Pediatrics, HU Puerta de Hierro, Majadahonda, Madrid, Spain; 5 Head of digital accessibility and inclusive design, Bankinter, Madrid, Spain; 6 Department of Epidemiology and Public Health, Department of Medical Specialties and Public Health, Universidad Rey Juan Carlos, Madrid, Spain; 7 Centro de Salud Núñez Morgado, Madrid, Spain

**Keywords:** Eosinophilic esophagitis, Health-related quality of life, Disease-specific questionnaire, Validation, PedsQL-EoE

## Abstract

**Background:**

The Paediatric Eosinophilic Oesophagitis Module (PedsQL-EoE) was developed in English as a valid and reliable questionnaire to assess health-related quality of life (HRQoL) in children with EoE. The aim of this study was to evaluate the validity and reliability of the PedsQL-EoE that was previously adapted to Spanish by our group.

**Methods:**

This cross-sectional multicentre study was conducted in 36 paediatric gastroenterology units. Groups with and without dietary restrictions were studied separately. The PedsQL-EoE consists of 33 items divided into seven factors. Age-specific versions of the PedsQL-EoE were sent by e-mail to children and parents. Statistical analysis was used to study the questionnaire structure by means of exploratory factor analysis and interitem correlations. Confirmatory factor analysis (CFA) was applied to verify the proposed model as well as its psychometric properties through SMSR (standardized root mean square), outer loadings and R-square. To study construct validity and reliability, Cronbach´s alpha coefficient, convergent validity (AVE), discriminant validity (HTMT) and intraclass correlation coefficients (ICC) were used.

**Results:**

A total of 341 children and 394 parents participated with 307 matched answers. The median age was 12 years, and 75% were male. The questionnaire structure explained 68% and 66% of the total variance for parents and children, respectively. Five items showed negative correlations and were removed from the questionnaire. CFA applied to the new model supported the following construct: SMRS was less than 0.08, outer loadings measured above 0.5, and R^2^ explained more than 89% of the total variance. Once the modifications were performed, good internal consistency was demonstrated, with Cronbach’s alpha values > 0.7, AVE values > 0.5 and HTMT < 0.9 with good child/parent agreement (ICC = 0.80). The most robust model of the PedsQL-EoE module was formed by seven factors: Symptoms I (6 items), Symptoms II (4 items), Treatment (4 items), Worries (3 items), Communication (5 items), Food and Eating (3 items) and Food Feelings (3 items).

**Conclusions:**

The final PedsQL-EoE Module version, after the removal of five items, is a valid and reliable tool to be used in children with EoE. The Spanish validated version appears to be a useful instrument for measuring the impact of EoE on Spanish children´s quality of life.

**Supplementary Information:**

The online version contains supplementary material available at 10.1186/s12955-023-02211-0.

## Background

Eosinophilic oesophagitis (EoE) is a chronic oesophageal immune disease characterized by oesophageal dysfunction and eosinophilic infiltration of at least 15 eosinophils per high-power field [1]. Predominant symptoms in children are vomiting, regurgitation, abdominal pain, food refusal or failure to thrive; Dysphagia, retrosternal pain, and impaction are more common in adults. The older the children, the more similar the symptoms to adult are. [2–4]. Three EoE treatment options are available: proton pump inhibitors, swallowed corticosteroids, and diet [5]. EoE impacts the patient’s quality of life (QoL) due to its recurrent and persistent symptoms, repeated diagnostic procedures, and chronic treatment requirements [6]. QoL is particularly impaired in patients following diet treatment due to both the diet itself and its impact on the patient’s socialization [7, 8].

Health-related quality of life (HRQoL) is a multidimensional construct composed of items related to the patient’s health, such as physical, emotional and social factors [9]. HRQoL questionnaires, both generic and specific, are considered the best instruments for evaluating the QoL of chronically ill patients [10]. The Paediatric Eosinophilic Oesophagitis Quality of Life Module (PedsQL-EoE) is the only questionnaire specifically addressed to children with EoE [7, 11]. Since HRQoL questionnaires are only applicable to the population for which they were created, cross-cultural adaptations of the questionnaire are necessary for their use in a different language.

Cross-cultural adaptation and validation of an instrument are complex processes that go beyond a literal translation between languages. The aim is to maintain the construct meaning, the operational properties, its administration procedure, data collection and response calculations. In addition, equivalence in terms of reliability and validity should be always respected to ensure the effectiveness of the instrument´s measurement.

The aim of this study is to evaluate the psychometric properties of the Spanish Peds QoL EoE Module, previously adapted by our group following Mapi research trust recommendations [12].

## Methods

### Translation and content validation

The aim of the cross-cultural adaptation was to evaluate the syntax and semantics of the different items of the children’s and parents’ questionnaires and to make contributions to the development of the Spanish version of the questionnaire. 5 patients and 5 parents per age group were recruited through AEDESEO (Asociación Española de Esofagitis Eosinofílica), and from the paediatric gastroenterology clinics of the research team.

The procedure followed the international recommendations: direct translation, consensus version, reverse translation, new consensus version, individual cognitive interviews with parents and children and final version. [12].

### Validation

#### Participants

This was a cross-sectional multicentre study conducted in 36 hospitals located in several regions of Spain (Madrid, Catalonia, Basque Country, Castile La Mancha, Castile and Leon, Andalusia, Galicia, Aragon, Canary Islands). The sample consisted of patients aged 2 to 18 with an EoE diagnosis based on oesophageal dysfunction together with 15 or more eosinophils per high-power field in the biopsy [1, 5] regardless of the time since diagnosis or treatment received. Patients with chronic diseases were excluded except for those related to atopic conditions because of the frequent association between these diseases and EoE.

#### Procedures

Patients were recruited during ordinary visits to the paediatric gastroenterology clinics of the participating centres following a probabilistic sampling method. The patients and their parents were informed about the objective of the study and questionnaire completion, receiving it by e-mail. A code with the centre and recruitment order was assigned to each child‒parent pair to ensure confidentiality. To calculate the time required to complete the questionnaires, the researchers established a temporizer. The recruitment period extended from October 2020 to October 2021. The study protocol was approved by the Hospital Universitario de Fuenlabrada Ethical Committee (code of approval: 19–05). Informed consent was obtained before study registration.

#### Measures

The specific HRQoL PedsQL-EoE questionnaire consisted of 33 items divided into seven factors: “Symptoms I”, “Symptoms II”, “Treatment”, “Worry”, “Communication”, “Food and Eating”, and “Food Feelings”. The questionnaire included distinct versions for parents and children aged 5–7, 8–12, and 13–18 and another version for parents of children aged 2–4.

The responses marked by the patients or parents were transformed into a 5-category Likert scale (0–4) and then changed into an inverse numerical scale from 0 to 100. The PEdsQL-EoE Total Scale Score was calculated as the average of the number of items answered in the seven factors. The Symptoms Total Scale Score was calculated as the average of the items of Symptoms I and Symptoms II. The HRQoL was described as very poor (0–20 points); poor (21–40); neutral (41–60); good (61–80); and very good (> 81) [7, 10].

We chose the version related to the HRQoL in the last month prior to completion of the questionnaire because it is more sensitive to each patient's situation and to changes attributed to treatments [7, 10, 13, 14].

#### Statistical analysis

The validation of the psychometric properties of the adapted Spanish version of the Peds QL-EoE Module was conducted through the following phases of analysis:

- 1- Internal Consistency:

Reliability was calculated using Cronbach's alpha coefficient. A value between 0.70 and 0.95 was considered as good internal consistency [15].

- 2- Exploratory factor analysis (EFA).

Construct validity analysis was performed through EFA [16]. The following assumptions were considered:

- Sample size:

The sample size to confirm that a stable factor solution can be obtained is 5–10 times the number of items [17, 18] which is 165 in our study. For that reason and due to the differences in the structure of the different versions of the questionnaires by age group, we studied only the 8–12 and 13–18 age groups, whose distribution of items was comparable both in number and in the equivalence of statements (33 items).

(b) Data adequacy for exploratory factor analysis:

The Kaiser‒Meyer‒Olkin (KMO) test (> 0.8) and Bartlett’s test of sphericity (*p* < 0.05) were used to assess the pertinence of performing aa EFA [19, 20].

(c) Factor extraction model:

The unweighted least squares extraction method was applied with eigenvalues > 1 to avoid the nonnormality effect of the sample. Total explained variance > 0.6 was considered acceptable [21, 22]. We applied an oblique rotation with the Promax method because oblique rotations are preferable when items are correlated [17, 23]. Coefficients < 0.3 were removed due to a lack of significant contribution to that factor [24].

- 3- Item Analysis:

To identify items that were not well correlated with the other items of the scale, we employed an interitem analysis [24]. Items with negative correlations can negatively affect the validity and reliability of the scale [19]. Items with excessively high correlations (> 0.8) may be redundant [25].

- 4- Confirmatory factor analysis (CFA):

A CFA was performed to confirm the new model proposed through the EFA investigation [26]. Children’s and parents´ questionnaires were analysed depending on whether they had dietary treatment. The main statistics studied were as follows:

- The model’s goodness-of-fit was evaluated through the standardized root mean square residual (SRMR). A value < 0.05 is a good model fit; values between 0.05 and 0.08 provided an acceptable model fit [27, 28].
- To study final construct reliability and validity, we used Cronbach’s alpha coefficient (**> **0.70) and composite variability (**> **0.7); for convergent validity, we used average variance extracted (AVE) (> 0.5), and for discriminant validity, we used a heterotrait-monotrail ratio (HTMT) < 0.9 [15, 22, 29, 30].
- To evaluate the structural modelling, we use out loadings, (> 0.5), adjusted R-square (substantial 75%, moderate 50%, weak 25%) [26] and the f-square (small 0.02, medium 0.15, large 0.35) [31].

- 5- Intraclass correlation coefficient (ICC):

Once the proposal of the questionnaire´s modification was performed, interobserver agreement (parents-children) was calculated as the ICC and 95% CI (confidence interval) (> 0.8 excellent, 0.6–0.8 good, 0.4—0.6 moderate and < 0.4 poor) [32].

All statistical tests were performed using the software package SPSS 27 for Windows (SPSS Inc., Chicago, IL, USA). Smart PLS 4 software was applied to perform CFA [33, 34].

## Results

Regarding the qualitative investigation, in the direct translation process and reverse translation, the questionnaire for children aged 5–7 underwent more modifications to produce an easier terminology.

All parents and 86% of the children understood the questionnaire. One-third of the children and 60% of the parents suggested modifications to the questionnaire. Some children reported problems with the double negatives that can be understood in the opposite way in Spanish. In consultation with the authors of the questionnaire, it was decided to keep the original version of the questionnaire.

### Sample characteristics

A total of 579 families agreed to participate in the study; 441 (76,2%) answered the questionnaire. There were 307 matched answers, 87 surveys were answered only by parents and 34 surveys were responded only by children. As a result, surveys were completed by a total of 341 children and 394 parents. Surveys from children 2 to 4 years old were completed only by parents. By age group, the highest number of responses was obtained in the 13–18 group (45.6%). The number of patients per participating centre is shown in Additional file 1. Demographic and clinical data are shown in Table 1. The mean time taken to complete the questionnaire was 6.3 (SD 5.5) minutes for children and 7.2 (SD 13.6) minutes for parents.

**Table 1:** Demographic data, disease characteristics and treatments of the children survey participants

		*N* = 350	%
Age Group	2–4 years	9	2.6
	5–7 years	32	9.1
	8–12 years	149	42.6
	13–18 years	160	45.7
Sex	Male	256	73.1
	Female	94	26.8
Symptoms at onset	Dysphagia	208	59.4
	Food impaction	151	43.1
	Abdominal pain	150	42.8
	Nausea, vomiting, regurgitation	148	42.3
	Heartburn	111	31.7
	Food avoidance	94	26.8
	Hoarseness	68	19.4
	Chest pain	65	18.6
	Weight loss	35	10
	Hematemesis	5	1.4
	Endoscopy incidental finding	13	3.7
Treatment	PPI	106	30.3
	Corticosteroids	141	40.3
	Diet	65	18.3
	Other^a^	2	0.1
	No treatment	36	10.3

^a^Other: 1 Omalizumab; 1 Montelukast

### Reliability

Regarding the analysis of reliability of the original questionnaire, the Cronbach´s alpha coefficient of the EoE Module Total Scale Score was 0.89 and 0.92 for children and parents respectively, indicating good internal consistency (Additional file 2).

### Questionnaire structure

The KMO values were 0.78 for children and 0.83 for parents, and Bartlett’s test of sphericity was significant (*p* < 0.001), confirming a correlation between the items for parents and children.

The sample recruited for the EFA was 309 children between 8 and 18 years of age and 351 parents. The 2–4 and 5–7 age groups could not be studied as the number of patients recruited (9 and 32, respectively) did not reach the minimum needed.

The results obtained by oblique rotation reported a different number of latent factors for parents [7] and for children [8], which constituted 68% and 66% of the total variance explained respectively.

In both matrices, we found some variables with shared weights in several factors. In both the parents’ (Fe1, Fe2, Fe3, Fo4, Fo5) and children´s matrices (Fe1, Fe2, Fe3, Fo2, Fo4, Fo5), we found items of “Food and Eating” and “Food Feelings” mixed in the same factor. Additionally, items corresponding to “Treatment” (Tr3, Tr4 and Tr5) were mixed in the same factor with questions corresponding to “Worries” (Wo4, Wo5 and Wo6) that had a similar syntax (Tables 2 and 3).

**Table 2:** Proposed factor structure for PedsQL-EoE in children with item loadings

FACTOR								
	1	2	3	4	5	6	7	8
Fo4	0.900							
Fo5	0.899							
Fo2	0.806							
Fe3	0.766							
Fe2	0.729							
Fe1	0.303							
Wo5		0.811						
Wo6		0.787						
Tr4		0.737						
Tr5		0.707						
Wo4		0.694						
Tr3		0.671						
Co3			0.818					
Co2			0.811					
Co4			0.810					
Co5			0.710					
Co1			0.351					
SII3				0.869				
SII1				0.869				
SII2				0.856				
SII4				0.379				
SI1					0.606			
SI6					0.505			
SI2					0.505			
SI3					0.303			
SI5						0.784		
SI4						0.646		
Fo3						0.333		
Wo1							0.696	
Wo2							0.625	
Wo3							0.461	
Tr1								0.770
Tr2								0.539

**Table 3:** Proposed factor structure for PedsQL-EoE in parents with item loadings

FACTORS							
	1	2	3	4	5	6	7
Tr4	0.894						
Tr5	0.882						
Wo4	0.872						
Wo5	0.845						
Wo6	0.839						
Tr3	0.803						
Co3		0.931					
Co2		0.877					
Co5		0.724					
Co4		0.721					
Co1		0.606					0.342
Fe3			0.894				
Fe2			0.893				
Fo5			0.633				
Fo4			0.610			0.382	
Fe1			0.589			-0.303	
SII1				1.044			
SII2				0.817			
SII3				0.750			
SII4				0.511			
SI6				0.327			
SI5					0.796		
SI4					0.631		
SI3					0.542		
SI2					0.541		
SI1					0.407		
Fo3						0.587	
Tr1						0.520	
Fo2						0.442	
Tr2						0.387	
Wo3							0.760
Wo1							0.559
Wo2							0.312

In the parents´ matrix, items Fo2 and Fo3 were mixed with Tr1 and Tr2 that had nothing to do with each other from a semantic point of view. In the children´s factor loading matrix, item Fo3 was placed in the same factor as some “Symptom I” items (SI4 and SI5). In addition, an eighth factor appeared that consisted of only 2 items (Tr1 and Tr2). Finally, the items grouped under the factors "Communication", "Symptoms I" and "Symptoms II" coincided with those of the original questionnaire.

### Item analysis

The Inter-Item Correlation Matrix (Additional file 3) showed some values with negative correlations in both the children's and the parents' matrices, which suggests the need to assess the withdrawal of some items. It is worth highlighting the correlations between 3 items of the “Treatment” factor (Tr3, Tr4 and Tr5) from both questionnaires (children and parents) with the items of the “Worries” factor (Wo4, Wo5 and Wo6).

In summary, based on the results of the item analysis, we propose the following working hypotheses. First, we removed 3 items from one of the two factors, Worries or Treatment, that presented semantic similarity (Tr3, Tr4 and Tr5 or Wo4, Wo5 and Wo6). Second, we evaluated the items that appeared mixed in the same factor, that did not seem to have a semantic relationship and that presented negative correlations in the interitem matrix, such as Tr1 and Fo2.

### CFA

To verify the impact of the proposed changes, CFA was performed to recalculate the basic psychometric properties of the questionnaire and to compare them with the original version. In the proposed model, 5 items (Wo4, Wo5, Wo6, Tr1 and Fo2) were ultimately removed.

**Fig. 1:**
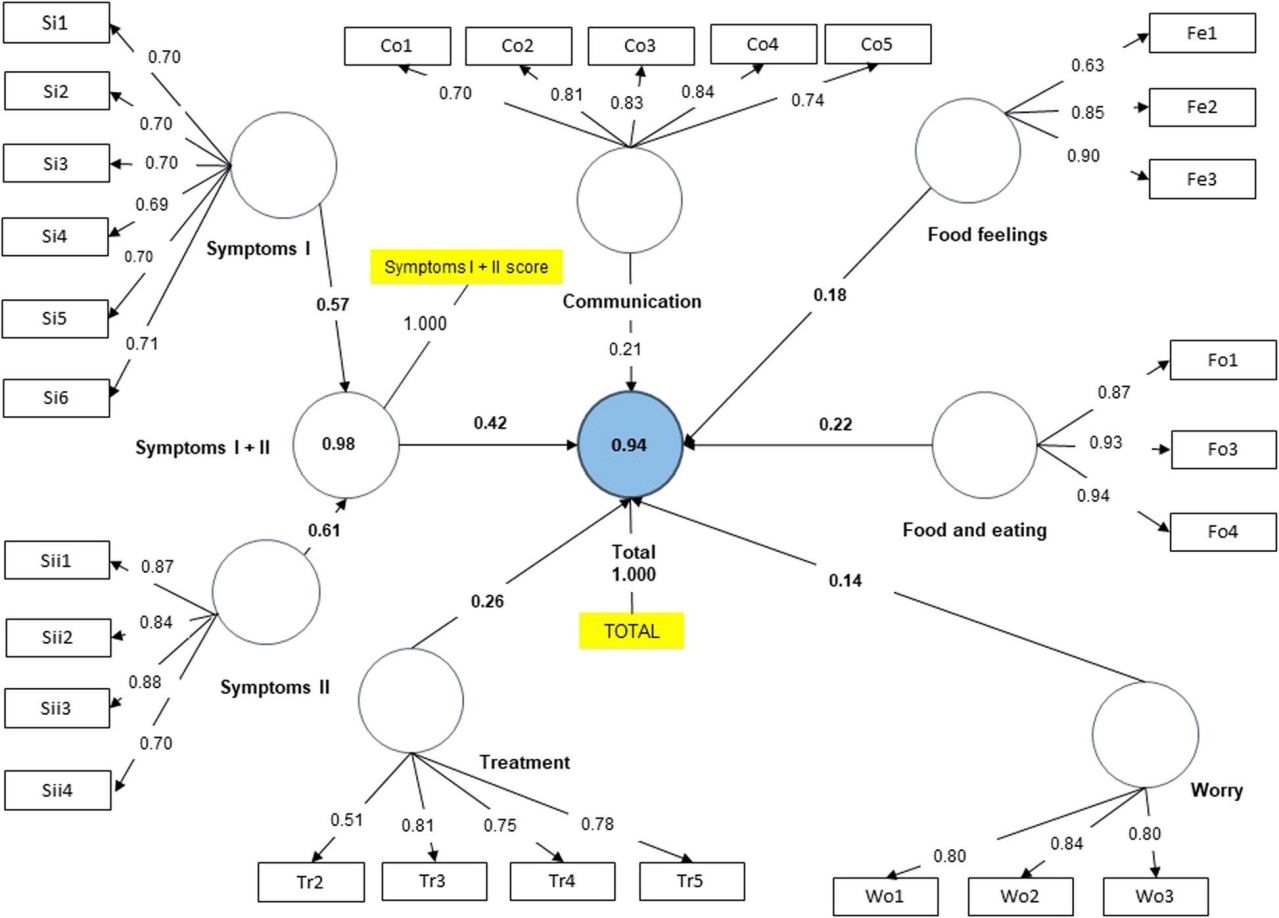
Final Model. Children under dietary treatment

**Fig. 2:**
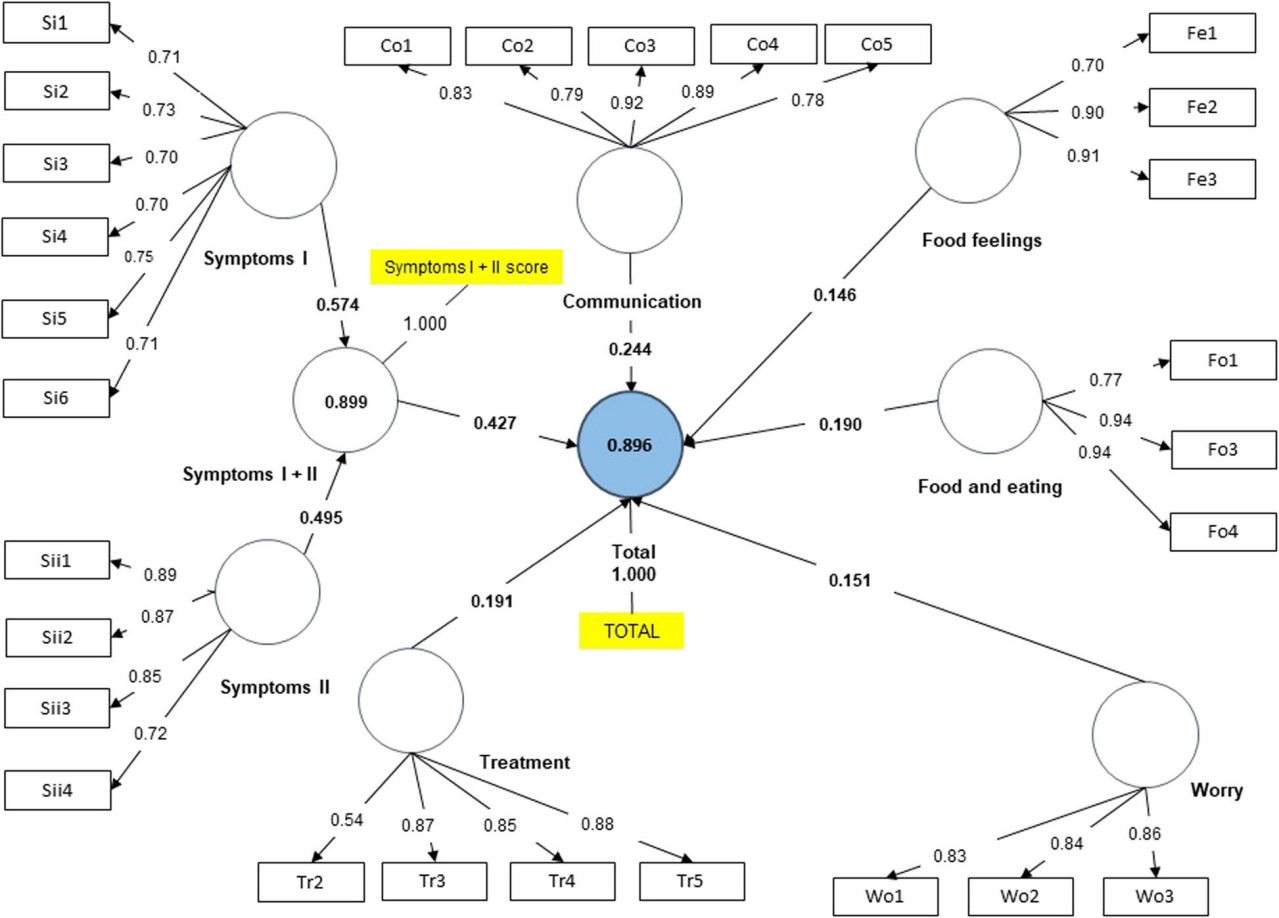
Final Model. Parents under dietary treatment

**Fig. 3:**
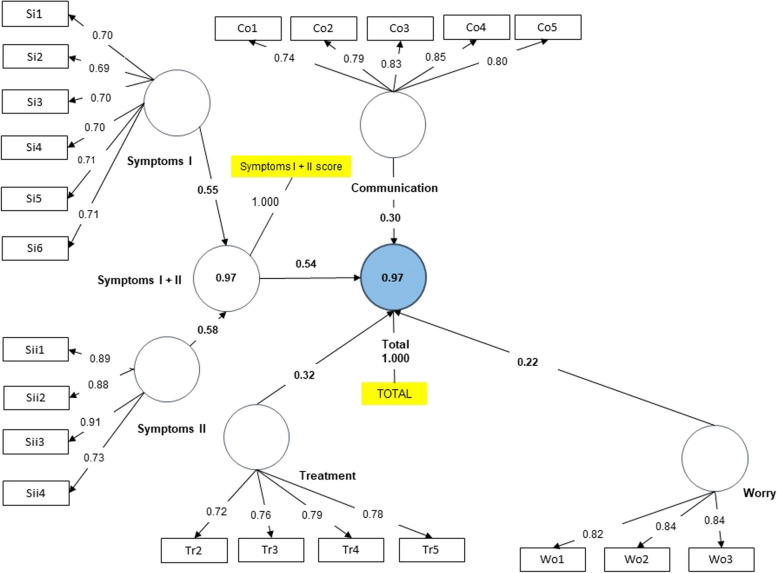
Final Model. Children under no dietary treatment

**Fig. 4:**
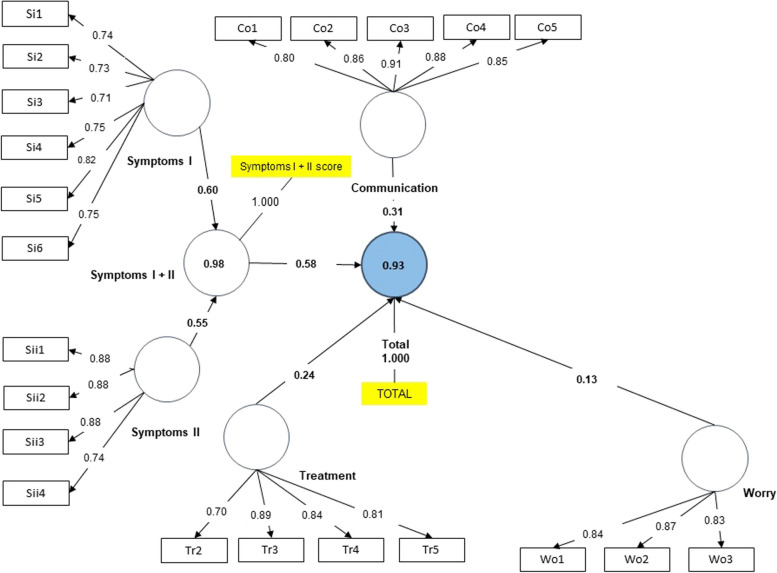
Final Model. Parents under no dietary treatment

For this purpose, two groups were studied: first, children undergoing dietary treatment and their parents; second, children without dietary treatment and their parents. For all models, SMRS values of less than 0.08 were obtained. However, the value for the group of children without dietary treatment was 0.086. The final models obtained with the CFAs are shown in Figs. 1, 2, 3 and 4. As depicted in the figures, values above 0.7 in the outer loadings show the absolute contributions between the items and the assigned factors, first level (EoE total scale, Symptoms I + II) and second level (Treatment, Worry, Food and Feelings and Food and Eating).

The adjusted R-square values indicated for the 4 groups that more than 89% of the variance was explained by the model for both the total and the symptoms scale. Regarding the f-square, the Worries factor had the lowest value in all groups, while the symptoms scale obtained a higher score in the large range measurement.

### Construct reliability and validity

Once the proposed modifications were made, the construct validity was recalculated using Cronbach's alpha coefficient. Values > 0.7 were obtained in all groups except for the “Treatment” in the group of children on dietary treatment (0.67). Composite variability reflected values > 0.7 for all dimensions in all 4 groups. AVE showed values > 0.5. Finally, HTMT estimator obtained values < the cut-off point of 0.9, as shown in Tables 4 and 5.

**Table 4:** Construct reliability and validity of the dimensions for the proposed version of the PedsQL-EoE in children

Children								
**Latent construct**	**Internal Consistency**				**Convergent Validity**		**Divergent Validity**	
	Cronbach’alpha^a^		Composite reliability^b^		Average Variance Extracted^c^		HTMT ratio^d^	
	Dietary treatment	Without dietary treatment	Dietary treatment	Without dietary treatment	Dietary treatment	Without dietary treatment	Dietary treatment	Without dietary treatment
Symptoms I	0.76	0.77	0.83	0.84	0.50	0.50	0.60	0.80
Symptoms II	0.82	0.88	0.88	0.90	0.66	0.74	0.65	0.69
Treatment	0.67	0.76	0.80	0.85	0.52	0.58	0.71	0.60
Worry	0.75	0.80	0.86	0.87	0.67	0.64	0.75	0.85
Communication	0.84	0.90	0.89	0.90	0.62	0.69	0.71	0.68
Food and Eating	0.90		0.94		0.87		0.69	
Food Feelings	0.71		0.84		0.64		0.78	

^a^Values above 0.7 indicate good internal consistency

^b^Values above 0.7 indicate good internal consistency

^c^Values above 0.5 indicate good convergent validity

4Values below 0.9 indicate a good divergent validity

**Table 5:** Construct reliability and validity of the dimensions for the proposed version of the PedsQL-EoE in parents

Parents								
**Latent construct**	**Internal consistency**				**Convergent Validity**		**Divergent Validity**	
	Cronbach’alpha^a^		Composite reliability^b^		Average Variance Extracted^c^		HTMT ratio^d^	
	Dietary treatment	Without dietary treatment	Dietary treatment	Without dietary treatment	Dietary treatment	Without dietary treatment	Dietary treatment	Without dietary treatment
Symptoms I	0.80	0.84	0.85	0.84	0.50	0.50	0.72	0.80
Symptoms II	0.85	0.86	0.90	0.90	0.70	0.74	0.60	0.69
Treatment	0.79	0.76	0.87	0.82	0.63	0.58	0.65	0.60
Worry	0.80	0.80	0.88	0.81	0.71	0.64	0.80	0.85
Communication	0.90	0.90	0.92	0.91	0.71	0.69	0.68	0.68
Food and Eating	0.86		0.92		0.78		0.73	
Food Feelings	0.78		0.87		0.70		0.66	

^a^Values above 0.7 indicate good internal consistency

^b^Values above 0.7 indicate good internal consistency

^c^Values above 0.5 indicate good convergent validity

^d^Values below 0.9 indicate a good divergent validity

The final Spanish Peds EoE QoL Module version is shown in Additional file 4.

### Intraclass correlation coefficient

Prior to calculating the ICC, a bias analysis of variance was performed using Student's t test for related samples. The ICC estimators between children and parents and their 95% CI were calculated based on a one-way random effects model. The ICC between children and parents, performed with modifications of the questionnaire, was 0.80 for the total scale with a range between 0.62–0.83, indicating good reliability (Table 6).

**Table 6:** Intraclass Correlation Coefficient (CI) between children and parents for the proposed version of PedsQL-EoE

		95% CI	
Scores	Intraclass Correlation	Lower Bound	Upper Bound
Total EoE Scale Score	0.80	0.74	0.84
Symptoms Scale	0.79	0.72	0.83
Symptoms I	0.75	0.68	0.80
Symptoms II	0.83	0.78	0.86
Treatment	0.62	0.52	0.70
Worry	0.74	0.66	0.79
Communication	0.68	0.53	0.70
Food and Eating	0.69	0.58	0.78
Food Feelings	0.76	0.67	0.82

## Discussion

To the best of our knowledge, this is the first study to report the translation and validation of the specific PedsQL-EoE Module in a language different from its original English version. Prior to validating the questionnaire, our group performed qualitative cross-cultural adaptation of the questionnaire according to internationally agreed-upon standards [12]. Our study revealed that once the appropriate modifications were made according to the psychometric study, the Spanish version of this questionnaire for children over 8 years old and their parents possessed adequate psychometric properties to assess HRQoL in Spanish children with EoE.

According to Cohen’s criteria, a questionnaire must be translated and validated in another language to be considered thoroughly valid [35]. Furthermore, using the same questionnaire that other authors have created allows performing comparative studies among patients from different countries suffering from the same disease.

Regarding feasibility, the response rate was good for both children and parents. Furthermore, the questionnaire was answered in less than ten minutes. Noticeably, a large sample of children diagnosed with EoE was obtained. Most recruited patients were distributed in the older age groups (aged 8–12 and 13–18), where the highest frequency of EoE diagnoses is accumulated. The predominance of males over females reflects findings reported by other research studies [1, 2].

In terms of internal reliability, our study exceeded the recommended alpha coefficient of 0.70, with values similar to those obtained by the original authors of the questionnaire. Moreover, in line with Franciosi et al. study [7, 11], the internal consistency of the “Symptoms II” factor was the lowest among all dimensions, together with “Symptoms I” in children aged 5–7 revealing the difficulty of children aged under eight in properly manifesting their symptoms.

To conduct the validity study by means of an initial EFA, data corresponding to parents and children over 8 years of age were selected since for younger ages, the number of patients obtained was not the minimum required to conduct this type of study [17, 18].

In both loading factor matrices, 7 factors were obtained for parents and 8 for children. The additional factor obtained for children was made up of three items, two corresponding to "Symptoms" and one to "Food and Feelings", which semantically had nothing to do with the other two items. Therefore, it was decided to keep the three items in their original factors. This also made it possible to homogenize the questionnaire for children and parents and to establish comparisons between them.

Continuing with the study of item adequacy, in Component 1 of the rotated component matrix for both parents and children, items corresponding to two different factors were mixed: Tr3, Tr4 and Tr5 with Wo4, Wo5 and Wo6. These items were semantically similar, being difficult for children and parents to differentiate between “I don´t like” and “I worry about”. According to Lloret-Segura et al., items that express a similar idea with slightly different wording may be redundant [21]. Moreover, in the interitem correlation matrix, these 6 items showed negative correlations two by two, Tr3/Wo4, Tr4/Wo5 and Tr5/Wo6, which supports their redundancy. It was proposed to remove the 3 items in either the "Treatment" or the "Worries" dimension. After performing different CFA models, it was concluded that the one with the best model fit was the one that retained the 3 items in the “Treatment” factor and removed them from the “Worries” factor. This decision avoided leaving the "Treatment" dimension with two questions, which was not an optimal configuration since the minimum number of items in a factor should be 3 [19].

Items Tr1 and Fo2 had no semantic relationship with the rest of the items of the factor in which they appeared in the factor extraction matrix and had negative correlations in the interitem matrix, so they were also removed from the questionnaire, as suggested by experts [22, 27, 28].

We also observed that the items corresponding to the factors "Food and Eating" and "Food Feelings" appeared together in both the parents' and the children's matrix, probably because it is difficult to distinguish between difficulty eating and the feeling that this limitation causes. However, because they were two different concepts, they were left in their original factors.

Once the proposed modifications were made (i.e., five items were removed: Wo4, Wo5, Wo6, Tr1 and Fo2), the results of the CFA should demonstrate a good fit of the proposed theoretical model as well as construct validity, convergent validity, discriminant validity and adequate reliability. When assessing the fit model, when we studied the outer loadings, good values were obtained for all items except for Tr1 (“Not wanting to take medicines”) in the dietary treatment group, may be because, generally, children on diets do not take medicines. In fact, in the questionnaire, practically all the surveyed answered “never.” Hence, in the diet group, this question was not very discriminating. According to Hu and Bentler, the SMRS estimator is the only criterion of overall goodness of fit [27]. SMRS values were adequate, except for children not following dietary treatment, with a value slightly above the cut-off. Furthermore, the adjusted R-square values indicated high explained variances for all 4 groups as more than 89% of variance was explained by the model for both the EoE total scale and the symptom scale. Regarding the impact of the removal of a factor on the questionnaire (F-square), the symptoms scale had the greatest impact, in the "large" range, while the removal of the "Worries" factor had the least impact according to Cohen's guidance [31].

Internal consistency was good, as evidenced by Cronbach's alpha values above 0.7 for all groups except that of the "Treatment" factor for children on a diet (0.68). Moreover, better Cronbach's alpha scores were obtained after the modifications than those initially obtained with the original questionnaire, which suggests improvement of the model.

Internal consistency was also demonstrated by the study of convergent validity through the AVE, whose values explained at least 50% of the variance of the respective indicators of each factor [32], and the values obtained from discriminant validity assessed through HTMT, all of which were lower than 0.9, indicating that the correlations between indicators that measured the same construct were higher than the correlations between indicators that measured different constructs [33].

Therefore, the Peds QoL EoE Module, in its Spanish version (Additional file 4), consists of the following factors with a total of 28 items: Symptoms I (6), Symptoms II (4), Treatment (4), Worries (3), Communication (5), Food and Eating (3) and Food Feelings (3).

Among the strengths of our study is the large number of patients recruited, which indicates that the age ranges in which EoE is most prevalent were well represented. In addition, the large group of participating centres allowed the sample to be representative of children diagnosed with EoE in Spain. However, our study presents some limitations. On the one hand, the low number of patients aged 2 to 4 and 5 to 7 years old prevented validation of the questionnaire in those age groups. On the other hand, the numbers of participants from the different centres were not homogeneous due to the different sizes of the participating centres, which could potentially constitute selection bias. Besides, sending the survey by e mail, prevents participants for asking any possible doubts. In addition, the final version of the questionnaire was not identical to the original version as 5 items had to be removed, which may make comparisons with populations in which the original questionnaire was applied difficult. Finally, it would be interesting to validate the questionnaire for the two younger groups. This leaves room for further investigation.

## Conclusions

The final PedsQL-EoE Module Spanish version, after the removal of five items, is a valid and reliable instrument for children 8–18 years old and their parents. It could be used to evaluate the HRQoL of Spanish children with EoE.

### Supplementary Information

**Additional file 1.** Spanish hospitals participating in the study.

**Additional file 2.** Cronbach’s alpha internal consistency reliability for children self-report and parents´ proxy-report by age.

**Additional file 3.** Children's and parents' Inter-Item correlation matrix.

**Additional file 4.** The final Spanish Peds EoE QoL Module version.

## Data Availability

The datasets analyzed during the current study are available from the corresponding author on reasonable request.

## Abbreviations

EoE: Eosinophilic Esophagitis
QoL: Quality of Life
HRQoL: Health Related Quality of Life
Peds QoL EoE Module: Pediatric Quality of Life Eosinophilic Esophagitis Module
AEDESEO: Spanish Association of Eosinophilic Esophagitis
EFA: Exploratory Factor Analysis
KMO: Kaiser–Meyer–Olkin
CFA: Confirmatory Factor Analysis
AVE: Average Variance Extracted
SMRS: Standardized Mean Root Square Residual
HTMT: Heterotrait Monotrail Ratio
ICC: Intraclass correlation coefficient
SD: Standard Deviation
SI: Symptoms I
SII: Symptoms II
Tr: Treatment
Wo: Worries
Co: Communication
Fo: Food and Eating
Fe: Food and Feelings
