# Supplementary material for: Psychometric evaluation of the Spanish version of the Pediatric Quality of Life Eosinophilic Esophagitis Questionnaire (Peds QL-EoE Module ™)
Source: Health Qual Life Outcomes. 2023 Dec 13;21:133. doi: 10.1186/s12955-023-02211-0 (PMC10717919; doi:10.1186/s12955-023-02211-0)
Supplement: Supplementary file 1 — Additional file 1. Spanish hospitals participating in the study. [file 12955_2023_2211_MOESM1_ESM.docx]

Additional File 1. Spanish hospitals participating in the study.

| PARTICIPATING HOSPITAL | NUMBER OF PATIENTS RECRUITED |
| --- | --- |
| Hospital Universitario de Fuenlabrada (Fuenlabrada) | 35 |
| Hospital Puerta de Hierro (Majadahonda) | 30 |
| H. Sant Joan de Deu. Spluges de Llobregat (Barcelona) | 29 |
| Hospital U. Infanta Elena (Valdemoro) | 19 |
| Hospital U Infanta Cristina (Parla) | 18 |
| Hospital Severo Ochoa (Leganés) | 16 |
| Hospital Fundación Alcorcón (Alcorcón) | 16 |
| H U Rey Juan Carlos (Móstoles) | 15 |
| H. U La Zarzuela (Madrid) | 15 |
| Hospital U de Guadalajara (Guadalajara) | 13 |
| H. HM Montepríncipe (Boadilla del Monte) | 12 |
| Hospital U Carlos Haya de Málaga (Málaga) | 12 |
| H Clínico San Carlos (Madrid) | 9 |
| H Miguel Servet (Zaragoza) | 9 |
| H. U Virgen del Rocío (Sevilla) | 8 |
| Hospital Gral. U de Ciudad Real (Ciudad Real) | 8 |
| H. la Mutua Tarrasa (Barcelona) | 7 |
| H. U Basurto (Bilbao) | 7 |
| H U general de Villalba (Villalba) | 7 |
| Hospital U. de Burgos (Burgos) | 6 |
| H.U Infanta Leonor Vallecas (Madrid) | 6 |
| Hospital General de Tomelloso (Ciudad Real) | 6 |
| H. U. Getafe (Getafe) | 6 |
| Hospital HM Sanchinarro (Madrid) | 5 |
| H. del Norte. Infanta Sofía (San Sebastián de los Reyes) | 5 |
| HU Ramón Y Cajal (Madrid) | 4 |
| Hospital U de Albacete (Albacete) | 4 |
| Hospital Clínico U. de Valladolid (Valladolid) | 4 |
| H. U. Fundación Jiménez Díaz (Madrid) | 4 |
| Complejo Hospitalario Universitario de Ourense (Ourense) | 4 |
| Hospital U Gregorio Marañón (Madrid) | 3 |
| Consorcio Corporación Sanitaria Parc Taulí (Sabadell) | 3 |
| H.U. Nuestra Señora de la Candelaria (Tenerife) | 2 |
| Hospital U de Valme (Sevilla) | 2 |
| H. Universitario de Canarias (Tenerife) | 1 |
| Hospital Santa Caterina - Salt (Girona) | * |

*The survey was answered only by parents
