# Supplementary material for: Psychometric evaluation of the Spanish version of the Pediatric Quality of Life Eosinophilic Esophagitis Questionnaire (Peds QL-EoE Module ™)
Source: Health Qual Life Outcomes. 2023 Dec 13;21:133. doi: 10.1186/s12955-023-02211-0 (PMC10717919; doi:10.1186/s12955-023-02211-0)
Supplement: Supplementary file 2 — Additional file 2. Cronbach’s alpha internal consistency reliability for children self-report and parents´ proxy-report by age. [file 12955_2023_2211_MOESM2_ESM.docx]

Additional file 2. Cronbach’s alpha internal consistency reliability for children self-report and parents´ proxy-report by age

| EoE Module Scales |  | Age group (years) | | | | |
| --- | --- | --- | --- | --- | --- | --- |
|  | **N** | **(2-4)** | **(5-7)** | **(8-12)** | **(13-18)** | **Total** |
| **Children Self report** | **341** | **N=0** | **N=32** | **N=149** | **N=160** | **N=341** |
| EoE Module Total Scale Score | 341 | NA | 0.91 | 0.87 | 0.91 | 0.89 |
| Symptoms Total Scale Score | 341 | NA | 0.76 | 0.81 | 0.84 | 0.82 |
| Symptoms I | 341 | NA | 0.65 | 0.75 | 0.77 | 0.76 |
| Symptoms II | 341 | NA | 0.64 | 0.83 | 0.83 | 0.83 |
| Treatment | 341 | NA | 0.74 | 0.60 | 0.69 | 0.64 |
| Worry | 341 | NA | 0.74 | 0.75 | 0.82 | 0.78 |
| Communication | 341 | NA | 0.81 | 0.82 | 0.87 | 0.84 |
| Food and Eating | 206 | NA | 0.84 | 0.76 | 0.87 | 0.82 |
| Food Feelings | 206 | NA | 0.73 | 0.70 | 0.70 | 0.71 |
| **Parent Proxy-Report** |  | **N=9** | **N=33** | **N=159** | **N=192** | **N=393** |
| EoE Module Total Scale Score | 394 | 0.71 | 0.82 | 0.90 | 0.93 | 0.92 |
| Symptoms Total Scale Score | 394 | 0.76 | 0.80 | 0.83 | 0.87 | 0.86 |
| Symptoms I | 394 | 0.61 | 0.70 | 0.79 | 0.83 | 0.81 |
| Symptoms II | 394 | 0.60 | 0.77 | 0.85 | 0.83 | 0.84 |
| Treatment | 394 | 0.83 | 0.74 | 0.66 | 0.78 | 0.72 |
| Worry | 394 | 0.73 | 0.78 | 0.81 | 0.80 | 0.80 |
| Communication | 385 | NA* | 0.91 | 0.91 | 0.90 | 0.91 |
| Food and Eating | 244 | 0.93 | 0.69 | 0.81 | 0.83 | 0.82 |
| Food Feelings | 244 | 0.73 | 0.74 | 0.78 | 0.76 | 0.77 |

^*^NA: Not applicable
