# Supplementary material for: Psychometric evaluation of the Spanish version of the Pediatric Quality of Life Eosinophilic Esophagitis Questionnaire (Peds QL-EoE Module ™)
Source: Health Qual Life Outcomes. 2023 Dec 13;21:133. doi: 10.1186/s12955-023-02211-0 (PMC10717919; doi:10.1186/s12955-023-02211-0)
Supplement: Supplementary file 3 — Additional file 3. Children's and parents' Inter-Item correlation matrix. [file 12955_2023_2211_MOESM3_ESM.docx]

Additional file 3. Children's and parents' Inter-Item correlation matrix

|  | | | | | | | | | | | | | | | | | | | | | | | | |
| --- | --- | --- | --- | --- | --- | --- | --- | --- | --- | --- | --- | --- | --- | --- | --- | --- | --- | --- | --- | --- | --- | --- | --- | --- |
| **Children** | | | | | | | | | | | |  | **Parents** | | | | | | | | | | | |
|  | **Tr1** | **Tr2** | **Tr3** | **Tr4** | **Tr5** | **Wo1** | **Wo2** | **Wo3** | **Wo4** | **Wo5** | **Wo6** |  | **Tr1** | **Tr2** | **Tr3** | **Tr4** | **Tr5** | **Wo1** | **Wo2** | **Wo3** | **Wo4** | **Wo5** | **Wo6** |  |
| **SI.1** | 0,21 | 0,09 | 0,02 | **-0,02** | **-0,03** | 0,08 | 0,13 | 0,22 | 0,09 | 0,03 | 0,00 |  | 0,11 | 0,13 | 0,12 | 0,09 | 0,13 | 0,32 | 0,40 | 0,27 | 0,07 | 0,12 | 0,16 | **SI.1** |
| **SI.2** | 0,23 | 0,03 | 0,01 | 0,04 | **-0,04** | 0,10 | 0,19 | 0,19 | 0,03 | 0,01 | 0,07 |  | 0,16 | 0,12 | 0,02 | 0,04 | 0,02 | 0,26 | 0,28 | 0,19 | 0,08 | 0,10 | 0,08 | **SI.2** |
| **SI.3** | 0,26 | 0,20 | 0,09 | **-0,05** | 0,04 | 0,05 | 0,11 | 0,09 | 0,08 | 0,02 | 0,10 |  | 0,13 | 0,18 | 0,12 | 0,14 | 0,16 | 0,19 | 0,27 | 0,15 | 0,18 | 0,20 | 0,22 | **SI.3** |
| **SI.4** | 0,11 | 0,12 | 0,13 | 0,00 | 0,06 | 0,06 | 0,13 | 0,16 | 0,14 | 0,07 | 0,06 |  | 0,06 | 0,19 | 0,16 | 0,09 | 0,14 | 0,18 | 0,42 | 0,22 | 0,08 | 0,10 | 0,16 | **SI.4** |
| **SI.5** | 0,26 | 0,16 | 0,01 | 0,00 | 0,05 | 0,14 | 0,19 | 0,17 | 0,01 | **-0,02** | 0,00 |  | 0,20 | 0,15 | 0,15 | 0,12 | 0,10 | 0,18 | 0,35 | 0,25 | 0,12 | 0,21 | 0,15 | **SI.5** |
| **SI.6** | 0,11 | 0,01 | **-0,06** | **-0,05** | **-0,07** | 0,01 | 0,04 | 0,13 | 0,03 | 0,05 | **-0,01** |  | 0,15 | 0,11 | 0,19 | 0,19 | 0,16 | 0,21 | 0,35 | 0,31 | 0,23 | 0,21 | 0,19 | **SI.6** |
| **SII.1** | 0,11 | 0,07 | 0,16 | 0,06 | 0,07 | 0,16 | 0,23 | 0,23 | 0,28 | 0,12 | 0,11 |  | 0,00 | 0,08 | 0,11 | 0,14 | 0,14 | 0,26 | 0,43 | 0,26 | 0,10 | 0,15 | 0,16 | **SII.1** |
| **SII.2** | 0,19 | 0,07 | 0,04 | **-0,06** | 0,04 | 0,05 | 0,25 | 0,22 | 0,17 | 0,01 | 0,06 |  | 0,13 | 0,09 | 0,06 | 0,09 | 0,10 | 0,27 | 0,49 | 0,30 | 0,02 | 0,12 | 0,11 | **SII.2** |
| **SII.3** | 0,12 | 0,13 | 0,13 | 0,03 | 0,11 | 0,11 | 0,30 | 0,17 | 0,18 | 0,11 | 0,14 |  | 0,06 | 0,05 | 0,11 | 0,09 | 0,12 | 0,27 | 0,37 | 0,25 | 0,13 | 0,16 | 0,16 | **SII.3** |
| **SII.4** | 0,10 | 0,21 | 0,23 | 0,17 | 0,15 | 0,13 | 0,17 | 0,16 | 0,20 | 0,21 | 0,21 |  | 0,00 | 0,11 | 0,07 | 0,17 | 0,17 | 0,30 | 0,33 | 0,27 | 0,12 | 0,21 | 0,18 | **SII.4** |
| **Tr1** |  | 0,43 | **-0,12** | **-0,16** | **-0,06** | 0,18 | 0,24 | 0,10 | **-0,01** | **-0,06** | 0,00 |  |  | 0,33 | 0,02 | **-0,08** | **-0,06** | 0,13 | 0,11 | 0,14 | 0,00 | **-0,07** | **-0,07** | **Tr1** |
| **Tr2** | 0,43 |  | 0,19 | 0,07 | 0,13 | 0,21 | 0,27 | 0,22 | 0,24 | 0,11 | 0,12 |  | 0,33 |  | 0,33 | 0,22 | 0,29 | 0,20 | 0,12 | 0,10 | 0,27 | 0,21 | 0,27 | **Tr2** |
| **Tr3** | **-0,12** | 0,19 |  | 0,56 | 0,52 | 0,16 | 0,25 | 0,22 | **0,65** | 0,45 | 0,46 |  | 0,02 | 0,33 |  | 0,65 | 0,70 | 0,24 | 0,27 | 0,28 | **0,82** | **0,59** | **0,64** | **Tr3** |
| **Tr4** | **-0,16** | 0,07 | 0,56 |  | 0,56 | 0,15 | 0,19 | 0,19 | 0,46 | **0,69** | 0,42 |  | **-0,08** | 0,22 | 0,65 |  | 0,70 | 0,27 | 0,26 | 0,26 | **0,67** | **0,90** | **0,65** | **Tr4** |
| **Tr5** | **-0,06** | 0,13 | 0,52 | 0,56 |  | 0,10 | 0,14 | 0,18 | 0,48 | 0,43 | **0,68** |  | **-0,06** | 0,29 | 0,70 | 0,70 |  | 0,24 | 0,29 | 0,27 | **0,72** | **0,65** | **0,94** | **Tr5** |
| **Wo1** | 0,18 | 0,21 | 0,16 | 0,15 | 0,10 |  | 0,53 | 0,45 | 0,30 | 0,32 | 0,26 |  | 0,13 | 0,20 | 0,24 | 0,27 | 0,24 |  | 0,51 | 0,61 | 0,36 | 0,38 | 0,31 | **Wo1** |
| **Wo2** | 0,24 | 0,27 | 0,25 | 0,19 | 0,14 | 0,53 |  | 0,50 | 0,28 | 0,19 | 0,25 |  | 0,11 | 0,12 | 0,27 | 0,26 | 0,29 | 0,51 |  | 0,60 | 0,26 | 0,29 | 0,30 | **Wo2** |
| **Wo3** | 0,10 | 0,22 | 0,22 | 0,19 | 0,18 | 0,45 | 0,50 |  | 0,38 | 0,22 | 0,23 |  | 0,14 | 0,10 | 0,28 | 0,26 | 0,27 | 0,61 | 0,60 |  | 0,29 | 0,33 | 0,32 | **Wo3** |
| **Wo4** | **-0,01** | 0,24 | **0,65** | 0,46 | 0,48 | 0,30 | 0,28 | 0,38 |  | 0,64 | 0,66 |  | 0,00 | 0,27 | **0,82** | **0,67** | **0,72** | 0,36 | 0,26 | 0,29 |  | 0,70 | 0,73 | **Wo4** |
| **Wo5** | **-0,06** | 0,11 | 0,45 | **0,69** | 0,43 | 0,32 | 0,19 | 0,22 | 0,64 |  | 0,68 |  | **-0,07** | 0,21 | **0,59** | **0,90** | **0,65** | 0,38 | 0,29 | 0,33 | 0,70 |  | 0,69 | **Wo5** |
| **Wo6** | 0,00 | 0,12 | 0,46 | 0,42 | 0,68 | 0,26 | 0,25 | 0,23 | 0,66 | 0,68 |  |  | **-0,07** | 0,27 | **0,64** | **0,65** | **0,94** | 0,31 | 0,30 | 0,32 | 0,73 | 0,69 |  | **Wo6** |
| **Co1** | 0,25 | 0,28 | 0,11 | 0,10 | 0,25 | 0,31 | 0,37 | 0,46 | 0,16 | 0,06 | 0,19 |  | 0,16 | 0,16 | 0,31 | 0,24 | 0,25 | 0,41 | 0,45 | **0,66** | 0,24 | 0,25 | 0,25 | **Co1** |
| **Co2** | 0,22 | 0,21 | 0,18 | 0,08 | 0,09 | 0,26 | 0,35 | 0,43 | 0,29 | 0,13 | 0,16 |  | 0,17 | 0,22 | 0,25 | 0,22 | 0,18 | 0,16 | 0,30 | 0,27 | 0,19 | 0,21 | 0,14 | **Co2** |
| **Co3** | 0,14 | 0,18 | 0,30 | 0,18 | 0,21 | 0,30 | 0,33 | 0,44 | 0,37 | 0,15 | 0,22 |  | 0,17 | 0,14 | 0,29 | 0,26 | 0,25 | 0,23 | 0,46 | 0,49 | 0,24 | 0,26 | 0,21 | **Co3** |
| **Co4** | 0,18 | 0,23 | 0,24 | 0,14 | 0,21 | 0,31 | 0,35 | **0,56** | 0,40 | 0,19 | 0,17 |  | 0,16 | 0,16 | 0,30 | 0,25 | 0,27 | 0,33 | 0,44 | 0,60 | 0,24 | 0,28 | 0,26 | **Co4** |
| **Co5** | 0,12 | 0,26 | 0,30 | 0,23 | 0,22 | 0,17 | 0,26 | 0,32 | 0,40 | 0,30 | 0,22 |  | 0,25 | 0,22 | 0,35 | 0,26 | 0,28 | 0,20 | 0,39 | 0,46 | 0,24 | 0,26 | 0,27 | **Co5** |
| **Fo2** | 0,13 | 0,24 | 0,16 | 0,12 | 0,19 | 0,37 | 0,16 | 0,18 | 0,19 | 0,19 | 0,18 |  | 0,20 | 0,27 | 0,29 | 0,21 | 0,32 | 0,40 | 0,27 | 0,26 | 0,34 | 0,23 | 0,34 | **Fo2** |
| **Fo3** | 0,19 | 0,12 | 0,05 | 0,01 | **-0,01** | 0,12 | 0,22 | 0,14 | 0,04 | **-0,06** | **-0,08** |  | 0,24 | 0,22 | 0,12 | 0,08 | 0,03 | 0,25 | 0,11 | 0,18 | 0,16 | 0,12 | 0,08 | **Fo3** |
| **Fo4** | 0,21 | 0,27 | 0,14 | 0,07 | 0,21 | 0,37 | 0,32 | 0,30 | 0,17 | 0,12 | 0,17 |  | 0,19 | 0,38 | 0,39 | 0,34 | 0,41 | 0,46 | 0,37 | 0,33 | 0,46 | 0,37 | 0,44 | **Fo4** |
| **Fo5** | 0,19 | 0,27 | 0,16 | 0,12 | 0,20 | 0,30 | 0,29 | 0,26 | 0,16 | 0,15 | 0,15 |  | 0,15 | 0,30 | 0,41 | 0,39 | 0,45 | 0,45 | 0,41 | 0,44 | 0,43 | 0,41 | 0,47 | **Fo5** |
| **Fe1** | **-0,06** | 0,09 | 0,28 | 0,28 | 0,20 | 0,26 | 0,19 | 0,09 | 0,28 | 0,29 | 0,28 |  | **-0,04** | 0,08 | 0,20 | 0,22 | 0,32 | 0,23 | 0,21 | 0,23 | 0,24 | 0,26 | 0,34 | **Fe1** |
| **Fe2** | 0,11 | 0,24 | 0,19 | 0,08 | 0,22 | 0,37 | 0,24 | 0,25 | 0,26 | 0,17 | 0,25 |  | 0,11 | 0,25 | 0,22 | 0,23 | 0,29 | 0,45 | 0,31 | 0,39 | 0,34 | 0,30 | 0,35 | **Fe2** |
| **Fe3** | 0,12 | 0,30 | 0,23 | 0,17 | 0,29 | 0,36 | 0,21 | 0,22 | 0,30 | 0,28 | 0,29 |  | 0,10 | 0,23 | 0,23 | 0,24 | 0,29 | 0,44 | 0,31 | 0,41 | 0,32 | 0,30 | 0,35 | **Fe3** |
