# Supplementary material for: Psychometric evaluation of the Spanish version of the Pediatric Quality of Life Eosinophilic Esophagitis Questionnaire (Peds QL-EoE Module ™)
Source: Health Qual Life Outcomes. 2023 Dec 13;21:133. doi: 10.1186/s12955-023-02211-0 (PMC10717919; doi:10.1186/s12955-023-02211-0)
Supplement: Supplementary file 4 — Additional file 4. The final Spanish Peds EoE QoL Module version. [file 12955_2023_2211_MOESM4_ESM.docx]

ID# __________________________

Fecha:________________________

**PedsQL**™ Módulo de Esofagitis Eosinofílica

Versión estándar 3.0

**CUESTIONARIO para ADOLESCENTES** (**de** **13 a 18 años**)

**INSTRUCCIONES**

Los/as adolescentes con Esofagitis Eosinofílica (EEo) a veces tienen problemas especiales. Dinos **hasta qué punto estas cosas han sido un problema** para ti durante el ***ÚLTIMO MES,*** marcando con un círculo:

**0** si **nunca** es un problema

**1** si **casi nunca** es un problema

**2** si **a veces** es un problema

**3** si **a menudo** es un problema

**4** si **casi siempre** es un problema

En este cuestionario no existen respuestas correctas o incorrectas.

Consúltanos si no entiendes alguna pregunta.

PedsQL 3.0 - (13-18) No se puede reproducir sin permiso Copyright © 1998 JW Varni, Ph.D. Todos los derechos reservados 4/2/2012

PedsQL 2 *Dinos hasta qué punto en el* ***ÚLTIMO mes*** *estas cosas han sido un* problema *para ti:*

| **MIS SÍNTOMAS** **I** ***(tengo problemas porque…)*** | **Nunca** | **Casi** **Nunca** | **A veces** | **A menudo** | **Casi** **Siempre** |
| --- | --- | --- | --- | --- | --- |
| 1. Tengo molestias o dolor en el pecho | 0 | 1 | 2 | 3 | 4 |
| 2. Me quema el pecho, la boca o la garganta (noto acidez) | 0 | 1 | 2 | 3 | 4 |
| 3. Tengo dolor de estómago o de tripa | 0 | 1 | 2 | 3 | 4 |
| 4. Tengo vómitos | 0 | 1 | 2 | 3 | 4 |
| 5. Siento que tengo ganas de vomitar, pero no lo hago (náuseas) | 0 | 1 | 2 | 3 | 4 |
| 6. Cuando como algo, se me sube a la garganta | 0 | 1 | 2 | 3 | 4 |

| **MIS SÍNTOMAS** **II** ***(tengo problemas porque…)*** | **Nunca** | **Casi** **Nunca** | **A veces** | **A menudo** | **Casi** **Siempre** |
| --- | --- | --- | --- | --- | --- |
| 1. Tengo problemas para tragar | 0 | 1 | 2 | 3 | 4 |
| 2. Siento como si la comida se me quedase atascada en la garganta o el pecho | 0 | 1 | 2 | 3 | 4 |
| 3. Necesito beber para ayudarme a tragar la comida | 0 | 1 | 2 | 3 | 4 |
| 4. Necesito más tiempo para comer que los chicos/as de mi edad | 0 | 1 | 2 | 3 | 4 |

| **MI TRATAMIENTO** ***(tengo problemas porque…)*** | **Nunca** | **Casi** **Nunca** | **A veces** | **A menudo** | **Casi** **Siempre** |
| --- | --- | --- | --- | --- | --- |
| 1. No quiero tomarme las medicinas | 0 | 1 | 2 | 3 | 4 |
| 2. No me gusta ir al médico | 0 | 1 | 2 | 3 | 4 |
| 3. No me gusta que me hagan endoscopias | 0 | 1 | 2 | 3 | 4 |
| 4. No me gusta que me hagan pruebas de alergia | 0 | 1 | 2 | 3 | 4 |

| **MIS PREOCUPACIONES** ***(tengo problemas porque…)*** | **Nunca** | **Casi** **Nunca** | **A veces** | **A menudo** | **Casi** **Siempre** |
| --- | --- | --- | --- | --- | --- |
| 1. Me preocupa tener esofagitis eosinofílica | 0 | 1 | 2 | 3 | 4 |
| 2. Me preocupa ponerme enfermo/a (vomitar, atascarme) delante de otras personas | 0 | 1 | 2 | 3 | 4 |
| 3. Me preocupa lo que los demás piensen de mi por tener esofagitis eosinofílica | 0 | 1 | 2 | 3 | 4 |


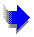
 **Pasa a la página siguiente** **para continuar con el resto de preguntas.** ¡**Gracias!.**

**Página siguiente.**

PedsQL 3.0 - (13-18) No se puede reproducir sin permiso Copyright © 1998 JW Varni, Ph.D. Todos los derechos reservados 4/2/2012

PedsQL 3 *Dinos hasta qué punto en el* ***ÚLTIMO mes*** *estas cosas han sido un* problema *para ti:*

| **CÓMO LES EXPLICO A LOS DEMÁS** ***(tengo problemas …)*** | **Nunca** | **Casi** **Nunca** | **A veces** | **A menudo** | **Casi** **Siempre** |
| --- | --- | --- | --- | --- | --- |
| 1. Para contarle a otras personas qué es una esofagitis eosinofílica | 0 | 1 | 2 | 3 | 4 |
| 2. Para hablar con mis padres sobre cómo me siento | 0 | 1 | 2 | 3 | 4 |
| 3. Para hablar con otros adultos sobre cómo me siento | 0 | 1 | 2 | 3 | 4 |
| 4. Para hablar con mis amigos/as sobre cómo me siento | 0 | 1 | 2 | 3 | 4 |
| 5. Para hablar con los médicos o enfermeras sobre cómo me siento | 0 | 1 | 2 | 3 | 4 |


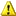
**¿Hay algún alimento al que eres alérgico o no lo puedes comer?**  Sí No *Si contestas* ***No****,* *deja las siguientes secciones* ***EN BLANCO.***

| **ALIMENTACIÓN Y COMER** ***(tengo problemas porque…)*** | **Nunca** | **Casi** **Nunca** | **A veces** | **A menudo** | **Casi** **Siempre** |
| --- | --- | --- | --- | --- | --- |
| 1. Es duro no poder comer algunos alimentos | 0 | 1 | 2 | 3 | 4 |
| 2. Es duro no poder comer las mismas cosas que mi familia | 0 | 1 | 2 | 3 | 4 |
| 3. Es duro no poder comer las mismas cosas que mis amigos/as | 0 | 1 | 2 | 3 | 4 |

| **MIS SENTIMIENTOS** ***(tengo problemas porque…)*** | **Nunca** | **Casi** **Nunca** | **A veces** | **A menudo** | **Casi** **Siempre** |
| --- | --- | --- | --- | --- | --- |
| 1. Me preocupa comer alimentos a lo que soy alérgico/a o que no debería comer | 0 | 1 | 2 | 3 | 4 |
| 2. Me enfada no poder comer alimentos a lo que soy alérgico/a o que no debería comer | 0 | 1 | 2 | 3 | 4 |
| 3. Me entristece no poder comer alimentos a los que soy alérgico/a o que no debería comer | 0 | 1 | 2 | 3 | 4 |


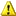
**¿Llevas sonda de alimentación en la nariz o en el estómago?** Sí No *Si contestas* ***No****,* *deja las siguientes secciones* ***EN BLANCO.***

| **MI SONDA DE ALIMENTACIÓN** ***(tengo problemas porque…)*** | **Nunca** | **Casi** **Nunca** | **A veces** | **A menudo** | **Casi** **Siempre** |
| --- | --- | --- | --- | --- | --- |
| ***COMPLETA LAS PREGUNTAS 1 Y 2 SÓLO SI LLEVAS SONDA DE ALIMENTACIÓN EN LA NARIZ O EN EL ESTÓMAGO*** | | | | | |
| 1. Me cuesta recordar usar la sonda de alimentación | 0 | 1 | 2 | 3 | 4 |
| 2. Me cuesta llevar la sonda de alimentación | 0 | 1 | 2 | 3 | 4 |

**¡Muchas gracias por participar!**

PedsQL 3.0 - (13-18) No se puede reproducir sin permiso Copyright © 1998 JW Varni, Ph.D. Todos los derechos reservados 4/2/2012

ID#__________________________

Fecha:________________________

**PedsQL**™ Módulo de Esofagitis Eosinofílica

Versión estándar 3.0

**CUESTIONARIO para PADRES de** **ADOLESCENTES** (**de** **13 a 18 años**)

**INSTRUCCIONES**

Los/as adolescentes con Esofagitis Eosinofílica (EEo) a veces tienen problemas especiales. Díganos **hasta qué punto estas cosas han sido un problema** para su hijo/a durante el ***ÚLTIMO MES,*** marcando con un círculo:

**0** si **nunca** es un problema

**1** si **casi nunca** es un problema

**2** si **a veces** es un problema

**3** si **a menudo** es un problema

**4** si **casi siempre** es un problema

En este cuestionario no existen respuestas correctas o incorrectas.

Consúltenos si no entiende alguna pregunta.

PedsQL 3.0 - Padres (13-18) No se puede reproducir sin permiso Copyright © 1998 JW Varni, Ph.D. Todos los derechos reservados 4/2/2012

PedsQL 2

*Díganos hasta qué punto en el* ***ÚLTIMO mes*** *estas cosas han sido un* problema *para su hijo/a:*

| **SÍNTOMAS** **I** ***(tiene problemas porque…)*** | **Nunca** | **Casi** **Nunca** | **A veces** | **A menudo** | **Casi** **Siempre** |
| --- | --- | --- | --- | --- | --- |
| 1. Tiene molestias o dolor en el pecho | 0 | 1 | 2 | 3 | 4 |
| 2. Le quema el pecho, la boca o la garganta (acidez) | 0 | 1 | 2 | 3 | 4 |
| 3. Tiene dolor de estómago o de tripa | 0 | 1 | 2 | 3 | 4 |
| 4. Vomita | 0 | 1 | 2 | 3 | 4 |
| 5. Siente como si fuera a vomitar, pero no lo hace (náuseas) | 0 | 1 | 2 | 3 | 4 |
| 6. Cuando come algo se le sube a la garganta | 0 | 1 | 2 | 3 | 4 |

| **SÍNTOMAS** **II** ***(tiene problemas porque…)*** | **Nunca** | **Casi** **Nunca** | **A veces** | **A**  **menudo** | **Casi** **Siempre** |
| --- | --- | --- | --- | --- | --- |
| 1. Tiene problemas para tragar | 0 | 1 | 2 | 3 | 4 |
| 2. Siente como si la comida se le quedase atascada en la garganta o el pecho | 0 | 1 | 2 | 3 | 4 |
| 3. Necesita beber para ayudar a tragar la comida | 0 | 1 | 2 | 3 | 4 |
| 4. Necesita más tiempo para comer | 0 | 1 | 2 | 3 | 4 |

| **TRATAMIENTO** ***(tiene problemas …)*** | **Nunca** | **Casi** **Nunca** | **A veces** | **A menudo** | **Casi** **Siempre** |
| --- | --- | --- | --- | --- | --- |
| 1. Porque no quiere tomarse las medicinas | 0 | 1 | 2 | 3 | 4 |
| 2. Por tener que ir al médico | 0 | 1 | 2 | 3 | 4 |
| 3. Para hacerse una endoscopia | 0 | 1 | 2 | 3 | 4 |
| 4. Para hacerse las pruebas de alergia | 0 | 1 | 2 | 3 | 4 |

| **PREOCUPACIONES** ***(tiene problemas con…)*** | **Nunca** | **Casi** **Nunca** | **A veces** | **A menudo** | **Casi** **Siempre** |
| --- | --- | --- | --- | --- | --- |
| 1. El hecho de tener esofagitis eosinofílica | 0 | 1 | 2 | 3 | 4 |
| 2. Ponerse enfermo/a (vomitar,atascarse) delante de otras personas | 0 | 1 | 2 | 3 | 4 |
| 3. Lo que los demás piensan de él/ella por tener esofagitis eosinofílica    4. Me preocupa ir al médico | 0 | 1 | 2 | 3 | 4 |


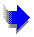


**Pase a la página siguiente** **para continuar con el resto de preguntas.** ¡**Gracias!.**

**Página siguiente.**

PedsQL 3.0 - Padres (13-18) No se puede reproducir sin permiso Copyright © 1998 JW Varni, Ph.D. Todos los derechos reservados 4/2/2012

PedsQL 3

*Díganos hasta qué punto en el* ***ÚLTIMO mes*** *estas cosas han sido un* problema *para su hijo/a:*

*?*

| **COMUNICACIÓN** ***(le ocasiona problemas…)*** | **Nunca** | **Casi** **Nunca** | **A veces** | **A menudo** | **Casi** **Siempre** |
| --- | --- | --- | --- | --- | --- |
| 1. Hablar con otras personas sobre la esofagitis eosinofílica | 0 | 1 | 2 | 3 | 4 |
| 2. Hablar con sus padres sobre cómo se siente  sobre cómo se siente | 0 | 1 | 2 | 3 | 4 |
| 3. Hablar con otros adultos sobre cómo se siente | 0 | 1 | 2 | 3 | 4 |
| 4. Hablar con sus amigos/as sobre cómo se siente | 0 | 1 | 2 | 3 | 4 |
| 5. Hablar con los médicos o enfermeras sobre cómo se siente | 0 | 1 | 2 | 3 | 4 |


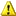
**¿Hay algún alimento al que tu hijo/a sea alérgico o no pueda comer?** Sí No *Si la respuesta es* ***No****,* *deja las siguientes secciones en blanco.*

| **ALIMENTACIÓN Y COMER *(tiene problemas con…)*** | **Nunca** | **Casi** **Nunca** | **A veces** | **A menudo** | **Casi** **Siempre** |
| --- | --- | --- | --- | --- | --- |
| 1. Seguir una dieta/restricciones alimentarias | 0 | 1 | 2 | 3 | 4 |
| 2. No poder comer las mismas cosas que su familia | 0 | 1 | 2 | 3 | 4 |
| 3. No poder comer las mismas cosas que sus amigos/as | 0 | 1 | 2 | 3 | 4 |

| **SENTIMIENTOS** ***(tiene problemas porque…)*** | **Nunca** | **Casi** **Nunca** | **A veces** | **A menudo** | **Casi** **Siempre** |
| --- | --- | --- | --- | --- | --- |
| 1. Le preocupa comer alimentos a los que es alérgico/a o que no debería comer | 0 | 1 | 2 | 3 | 4 |
| 2. Le enfada y/o le frustra no poder comer alimentos a lo que es alérgico/a o que no debería comer | 0 | 1 | 2 | 3 | 4 |
| 3. Le entristece no poder comer alimentos a lo que es alérgico/a o que no debería comer | 0 | 1 | 2 | 3 | 4 |


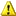
**¿Lleva tu hijo/a sonda de alimentación en la nariz o en el estómago?** Sí No *Si contestas* ***No****,* *deja las siguientes secciones* ***EN BLANCO.***

| **SONDA DE ALIMENTACION** ***(tiene problemas porque…)*** | **Nunca** | **Casi** **Nunca** | **A veces** | **A menudo** | **Casi** **Siempre** |
| --- | --- | --- | --- | --- | --- |
| ***COMPLETA LAS PREGUNTAS 1 Y 2 SÓLO SI TU HIJO/A LLEVA SONDA DE ALIMENTACIÓN EN LA NARIZ O EN EL ESTÓMAGO*** | | | | | |
| 1. Le cuesta recordar usar la sonda de alimentación | 0 | 1 | 2 | 3 | 4 |
| 2. Le cuesta llevar la sonda de alimentación | 0 | 1 | 2 | 3 | 4 |

**¡Muchas gracias por participar!**

PedsQL 3.0 - Padres (13-18) No se puede reproducir sin permiso Copyright © 1998 JW Varni, Ph.D. Todos los derechos reservados 4/2/2012

ID# __________________________

Fecha:________________________

**PedsQL**™ Módulo de Esofagitis Eosinofílica

Versión estándar 3.0

**CUESTIONARIO para NIÑOS/AS** (**de** **8 a 12 años**)

**INSTRUCCIONES**

Los niños/as con de Esofagitis Eosinofílica (EoE) a veces tienen problemas especiales. Dinos **hasta qué punto estas cosas han sido un problema** para ti durante el ***ÚLTIMO MES,*** marcando con un círculo:

**0** si **nunca** es un problema

**1** si **casi nunca** es un problema

**2** si **a veces** es un problema

**3** si **a menudo** es un problema

**4** si **casi siempre** es un problema

En este cuestionario no existen respuestas correctas o incorrectas.

Consúltanos si no entiendes alguna pregunta.

PedsQL 3.0 - (8-12) No se puede reproducir sin permiso Copyright © 1998 JW Varni, Ph.D. Todos los derechos reservados 4/2/2012

PedsQL 2 *Dinos hasta qué punto en el* ***ÚLTIMO mes*** *estas cosas han sido un* problema *para ti:*

| **MIS SÍNTOMAS** **I** ***(tengo problemas porque…)*** | **Nunca** | **Casi** **Nunca** | **A veces** | **A menudo** | **Casi** **Siempre** |
| --- | --- | --- | --- | --- | --- |
| 1. Tengo molestias o dolor en el pecho | 0 | 1 | 2 | 3 | 4 |
| 2. Me quema el pecho, la boca o la garganta (noto acidez) | 0 | 1 | 2 | 3 | 4 |
| 3. Tengo dolor de estómago o de tripa | 0 | 1 | 2 | 3 | 4 |
| 4. Tengo vómitos | 0 | 1 | 2 | 3 | 4 |
| 5. Siento que tengo ganas de vomitar, pero no lo hago (náuseas) | 0 | 1 | 2 | 3 | 4 |
| 6. Cuando como algo se me sube a la garganta | 0 | 1 | 2 | 3 | 4 |

| **MIS SÍNTOMAS** **II** ***(tengo problemas porque…)*** | **Nunca** | **Casi** **Nunca** | **A veces** | **A menudo** | **Casi** **Siempre** |
| --- | --- | --- | --- | --- | --- |
| 1. Tengo problemas para tragar | 0 | 1 | 2 | 3 | 4 |
| 2. Siento como si la comida se me quedase atascada en la garganta o el pecho | 0 | 1 | 2 | 3 | 4 |
| 3. Necesito beber para ayudarme a tragar la comida | 0 | 1 | 2 | 3 | 4 |
| 4. Necesito más tiempo para comer que otros niños/as de mi edad | 0 | 1 | 2 | 3 | 4 |

| **MI TRATAMIENTO** ***(tengo problemas porque…)*** | **Nunca** | **Casi** **Nunca** | **A veces** | **A menudo** | **Casi** **Siempre** |
| --- | --- | --- | --- | --- | --- |
| 1. No quiero tomarme las medicinas | 0 | 1 | 2 | 3 | 4 |
| 2. No me gusta ir al médico | 0 | 1 | 2 | 3 | 4 |
| 3. No me gusta que me hagan endoscopias | 0 | 1 | 2 | 3 | 4 |
| 4. No me gusta que me hagan pruebas de alergia | 0 | 1 | 2 | 3 | 4 |

| **MIS PREOCUPACIONES** ***(tengo problemas porque…)*** | **Nunca** | **Casi** **Nunca** | **A veces** | **A menudo** | **Casi** **Siempre** |
| --- | --- | --- | --- | --- | --- |
| 1. Me preocupa tener esofagitis eosinofílica | 0 | 1 | 2 | 3 | 4 |
| 2. Me preocupa ponerme enfermo/a (vomitar, atascarme..) delante de otras personas | 0 | 1 | 2 | 3 | 4 |
| 3. Me preocupa lo que los demás piensen de mí por tener esofagitis eosinofílica | 0    0 | 1    1 | 2 | 3 | 4 |


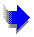


**Pasa a la página siguiente** **para continuar con el resto de preguntas.** ¡**Gracias!.**

**Página siguiente.**

PedsQL 3.0 - (8-12) No se puede reproducir sin permiso Copyright © 1998 JW Varni, Ph.D. Todos los derechos reservados 4/2/2012

PedsQL 3 *Dinos hasta qué punto en el* ***ÚLTIMO mes*** *estas cosas han sido un* problema *para ti:*

| **CÓMO LES EXPLICO A LOS DEMÁS** ***(tengo problemas porque…)*** | **Nunca** | **Casi** **Nunca** | **A veces** | **A menudo** | **Casi** **Siempre** |
| --- | --- | --- | --- | --- | --- |
| 1. Me cuesta contarles a otras personas qué es la esofagitis eosinofílica | 0 | 1 | 2 | 3 | 4 |
| 2. Me cuesta hablar con mis padres sobre cómo me siento | 0 | 1 | 2 | 3 | 4 |
| 3. Me cuesta hablar con otros adultos sobre cómo me siento | 0 | 1 | 2 | 3 | 4 |
| 4. Me cuesta hablar con mis amigos/as sobre cómo me siento | 0 | 1 | 2 | 3 | 4 |
| 5. Me cuesta hablar con los médicos o enfermeras sobre cómo me siento | 0 | 1 | 2 | 3 | 4 |


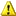
**¿Hay algún alimento al que eres alérgico/a o que no puedes comer?** Sí No

*Si contestas* ***No****,* *deja las siguientes secciones* ***EN BLANCO.***

| **ALIMENTACIÓN Y COMER** ***(tengo problemas porque…)*** | **Nunca** | **Casi** **Nunca** | **A veces** | **A menudo** | **Casi** **Siempre** |
| --- | --- | --- | --- | --- | --- |
| 1. Es difícil no poder comer algunos alimentos | 0 | 1 | 2 | 3 | 4 |
| 2. Es difícil no poder comer las mismas cosas que mi familia | 0 | 1 | 2 | 3 | 4 |
| 3. Es difícil no poder comer las mismas cosas que mis amigos/as | 0 | 1 | 2 | 3 | 4 |

| **MIS SENTIMIENTOS** ***(tengo problemas porque…)*** | **Nunca** | **Casi** **Nunca** | **A veces** | **A menudo** | **Casi** **Siempre** |
| --- | --- | --- | --- | --- | --- |
| 1. Me preocupa comer alimentos a lo que soy alérgico/a o que no debería comer | 0 | 1 | 2 | 3 | 4 |
| 2. Me enfada no poder comer alimentos a lo que soy alérgico/a o que no debería comer | 0 | 1 | 2 | 3 | 4 |
| 3. Me entristece no poder comer alimentos a lo que soy alérgico/a o que no debería comer | 0 | 1 | 2 | 3 | 4 |


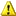
 **¿Llevas sonda de alimentación en la nariz o en el estómago?** Sí No

*Si contestas* ***No****,* *deja las siguientes secciones* ***EN BLANCO.***

| **MI SONDA DE ALIMENTACIÓN** ***(tengo problemas porque…)*** | **Nunca** | **Casi** **Nunca** | **A veces** | **A menudo** | **Casi** **Siempre** |
| --- | --- | --- | --- | --- | --- |
| ***COMPLETA LAS PREGUNTAS 1 Y 2 SÓLO SI LLEVAS SONDA DE ALIMENTACIÓN*** | | | | | |
| 1. Me cuesta recordar usar la sonda de alimentación | 0 | 1 | 2 | 3 | 4 |
| 2. Me cuesta llevar la sonda de alimentación | 0 | 1 | 2 | 3 | 4 |

**¡Muchas gracias por participar!**

PedsQL 3.0 - (8-12) No se puede reproducir sin permiso Copyright © 1998 JW Varni, Ph.D. Todos los derechos reservados 4/2/2012

ID# __________________________

Fecha:_________________________

**PedsQL**™ Módulo de Esofagitis Eosinofílica

Versión estándar 3.0

**CUESTIONARIO para PADRES** de **NIÑOS/AS** (**de** **8 a 12 años**)

**INSTRUCCIONES**

Los niños/as con Esofagitis Eosinofílica (EEo) a veces tienen problemas especiales. Díganos **hasta qué punto estas cosas han sido un problema** para su hijo/a durante el ***ÚLTIMO MES,*** marcando con un círculo:

**0** si **nunca** es un problema

**1** si **casi nunca** es un problema

**2** si **a veces** es un problema

**3** si **a menudo** es un problema

**4** si **casi siempre** es un problema

En este cuestionario no existen respuestas correctas o incorrectas.

Consúltenos si no entiende alguna pregunta.

PedsQL 3.0 - Padres (8-12) No se puede reproducir sin permiso Copyright © 1998 JW Varni, Ph.D.Todos los derechos reservados 4/2/2012

PedsQL 2

*Díganos hasta qué punto en el* ***ÚLTIMO mes*** *estas cosas han sido un* problema *para su hijo/a:*

| **SÍNTOMAS** **I** ***(tiene problemas porque…)*** | **Nunca** | **Casi** **Nunca** | **A veces** | **A menudo** | **Casi** **Siempre** |
| --- | --- | --- | --- | --- | --- |
| 1. Tiene molestias o dolor en el pecho | 0 | 1 | 2 | 3 | 4 |
| 2. Le quema el pecho, la boca o la garganta (acidez) | 0 | 1 | 2 | 3 | 4 |
| 3. Tiene dolor de estómago o de tripa | 0 | 1 | 2 | 3 | 4 |
| 4. Vomita | 0 | 1 | 2 | 3 | 4 |
| 5. Se siente como si fuera a vomitar, pero no lo hace (náuseas) | 0 | 1 | 2 | 3 | 4 |
| 6. Cuando come algo se le sube a la garganta | 0 | 1 | 2 | 3 | 4 |

| **SÍNTOMAS** **II** ***(tiene problemas porque…)*** | **Nunca** | **Casi** **Nunca** | **A veces** | **A menudo** | **Casi** **Siempre** |
| --- | --- | --- | --- | --- | --- |
| 1. Tiene problemas para tragar | 0 | 1 | 2 | 3 | 4 |
| 2. Siente como si la comida se le quedase atascada en la garganta o el pecho | 0 | 1 | 2 | 3 | 4 |
| 3. Necesita beber para ayudar a tragar la comida | 0 | 1 | 2 | 3 | 4 |
| 4. Necesita más tiempo para comer | 0 | 1 | 2 | 3 | 4 |

| **TRATAMIENTO** ***(tiene problemas …)*** | **Nunca** | **Casi** **Nunca** | **A veces** | **A menudo** | **Casi** **Siempre** |
| --- | --- | --- | --- | --- | --- |
| 1. Porque no quiere tomarse las medicinas | 0 | 1 | 2 | 3 | 4 |
| 2. Por tener que ir al médico | 0 | 1 | 2 | 3 | 4 |
| 3. Para hacerse una endoscopia | 0 | 1 | 2 | 3 | 4 |
| 4. Para hacerse pruebas de alergia | 0 | 1 | 2 | 3 | 4 |

| **PREOCUPACIONES** ***(tiene problemas con…)*** | **Nunca** | **Casi** **Nunca** | **A veces** | **A menudo** | **Casi** **Siempre** |
| --- | --- | --- | --- | --- | --- |
| 1. El hecho de tener esofagitis eosinofílica | 0 | 1 | 2 | 3 | 4 |
| 2. Ponerse enfermo/a (vómitos, atascarse) delante de otras personas | 0 | 1 | 2 | 3 | 4 |
| 3. Lo que los demás piensan de él/ella por tener esofagitis eosinofílica | 0 | 1 | 2 | 3 | 4 |


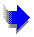


**Pase a la página siguiente** **para continuar con el resto de preguntas.** ¡**Gracias!.**

**Página siguiente.**

PedsQL 3.0 - Padres (8-12)No se puede reproducir sin permiso Copyright © 1998 JW Varni, Ph.D.Todos los derechos reservados4/2/2012

PedsQL3

*Díganos hasta qué punto en el* ***ÚLTIMO mes*** *estas cosas han sido un* problema *para su hijo/a:*

| **COMUNICACIÓN** ***(le ocasiona problemas…)*** | **Nunca** | **Casi** **Nunca** | **A veces** | **A menudo** | **Casi** **Siempre** |
| --- | --- | --- | --- | --- | --- |
| 1. Hablar a otras personas sobre la esofagitis eosinofílica | 0 | 1 | 2 | 3 | 4 |
| 2. Hablar con sus padres sobre cómo se siente | 0 | 1 | 2 | 3 | 4 |
| 3. Hablar con otros adultos sobre cómo se siente | 0 | 1 | 2 | 3 | 4 |
| 4. Hablar con sus amigos/as sobre cómo se siente | 0 | 1 | 2 | 3 | 4 |
| 5. Hablar con los médicos o enfermeras sobre cómo se siente | 0 | 1 | 2 | 3 | 4 |


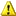
 **¿Hay algún alimento al que tu hijo/a sea alérgico o no pueda comer?** Sí No

*Si contestas* ***No****,* *deja las siguientes secciones en blanco.*

| **ALIMENTACIÓN Y COMER** ***(tiene problemas con…)*** | **Nunca** | **Casi** **Nunca** | **A veces** | **A menudo** | **Casi** **Siempre** |
| --- | --- | --- | --- | --- | --- |
| 1. Seguir una dieta/restricciones alimentarias | 0 | 1 | 2 | 3 | 4 |
| 3. No poder comer las mismas cosas que su familia | 0 | 1 | 2 | 3 | 4 |
| 4. No poder comer las mismas cosas que sus amigos/as | 0 | 1 | 2 | 3 | 4 |

| **SENTIMIENTOS** ***(tiene problemas porque…)*** | **Nunca** | **Casi** **Nunca** | **A veces** | **A menudo** | **Casi** **Siempre** |
| --- | --- | --- | --- | --- | --- |
| 1. Le preocupa comer alimentos a los que es alérgico/a o que no debería comer | 0 | 1 | 2 | 3 | 4 |
| 2. Le enfada y/o le frustra no poder comer alimentos a los que es alérgico/a o que no debería comer | 0 | 1 | 2 | 3 | 4 |
| 3. Le entristece no poder comer alimentos a los que es alérgico/a o que no debería comer | 0 | 1 | 2 | 3 | 4 |


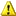
**¿Lleva tu hijo/a sonda de alimentación en la nariz o en el estómago?** Si No

*Si contestas* ***No****,* *deja las siguientes secciones* ***EN BLANCO.***

| **SONDA DE ALIMENTACIÓN** ***(tiene problemas porque…)*** | **Nunca** | **Casi** **Nunca** | **A veces** | **A menudo** | **Casi** **Siempre** |
| --- | --- | --- | --- | --- | --- |
| ***COMPLETA LAS PREGUNTAS 1 Y 2 SÓLO SI TU HIJO/A LLEVA SONDA DE ALIMENTACION EN LA NARIZ O EN EL ESTÓMAGO*** | | | | | |
| 1. Le cuesta recordar usar la sonda de alimentación | 0 | 1 | 2 | 3 | 4 |
| 2. Le cuesta llevar la sonda de alimentación | 0 | 1 | 2 | 3 | 4 |

**¡Muchas gracias por participar!**

PedsQL 3.0 - Padres (8-12)No se puede reproducir sin permiso Copyright © 1998 JW Varni, Ph.D.Todos los derechos reservados4/2/2012
